# Time- and Temperature-Dependent Luminescence of Manganese Ions in Ceramic Magnesium Aluminum Spinels

**DOI:** 10.3390/ma14020420

**Published:** 2021-01-16

**Authors:** Nicholas Khaidukov, Angela Pirri, Maria Brekhovskikh, Guido Toci, Matteo Vannini, Barbara Patrizi, Vladimir Makhov

**Affiliations:** 1 N. S. Kurnakov Institute of General and Inorganic Chemistry, 31 Leninskiy Prospekt, 119991 Moscow, Russia; khaiduk2@gmail.com (N.K.); mbrekh@mail.ru (M.B.); 2 Istituto di Fisica Applicata “N. Carrara”, Consiglio Nazionale delle Ricerche, via Madonna del Piano 10, Sesto Fiorentino, 50019 Florence, Italy; pirri@ifac.cnr.it; 3 Istituto Nazionale di Ottica, Consiglio Nazionale delle Ricerche, via Madonna del Piano 10, Sesto Fiorentino, 50019 Florence, Italy; guido.toci@ino.cnr.it (G.T.); matteo.vannini@ino.it (M.V.); barbara.patrizi@ino.cnr.it (B.P.); 4 P. N. Lebedev Physical Institute, 53 Leninskiy Prospekt, 119991 Moscow, Russia

**Keywords:** spinel, ceramics, photoluminescence, time-resolved spectroscopy, red-emitting Mn^4+^ phosphors, phosphor-converted LED

## Abstract

Samples of magnesium aluminum spinel ceramics doped with manganese ions were prepared by a high-temperature solid-state reaction method; their potential as red-emitting phosphors was analyzed using a time-resolved luminescence spectroscopy technique, from room temperature to 10 K. It was found that in the red spectral range, the luminescence spectra of manganese ions in the MgAl_2_O_4_ spinel showed a narrow band peaking at 651 nm due to the emission of Mn^4+^ and a broader emission band in the region of 675 ÷ 720 nm; the ratio of intensities for these bands depends on the synthesis conditions. By applying a special multi-step annealing procedure, the MgAl_2_O_4_:Mn^4+^ phosphor containing only tetravalent manganese ions, Mn^4+^, was synthesized. Broad-band far-red emission observed from MgAl_2_O_4_:Mn and Mg_1.25_Al_1.75_O_3.75_F_0.25_:Mn phosphors, prepared by a conventional method of a solid-state reaction, was interpreted as coming from Mn^3+^ ions.

## 1. Introduction

At present, the development of phosphors for application in phosphor-converted white Light Emitting Diodes (pc-WLEDs) is one of the most relevant areas of research in lighting technologies. In particular, since the introduction of the first commercial pc-WLEDs, a search for new efficient red light-emitting phosphors has been actively ongoing. Indeed, a significant contribution in the red region of the emission spectrum is highly required to obtain warm white light from pc-WLEDs based on the standard technology exploiting the combination of a blue LED chip and a converting yellow YAG:Ce^3+^-type phosphor [1]. A suitable phosphor should have significant absorption in the blue spectral range and emit in the red (i.e., in the 620 ÷ 650 nm range) [2]. In particular, in most commercial pc-WLEDs, some nitride compounds doped with Eu^2+^ rare earth ions are used as red phosphors [3]. However, the band width of the emission due to the Eu^2+^ 5d-4f electronic transition is relatively large, which results in partial emission outside the red region and, as a consequence, a decrease in the luminous efficacy of the red-emitting phosphor [3]. Mn^4+^-doped luminescent materials with narrow-band emission due to Mn^4+^ d-d electronic transition have been subjected to intensive studies in the last years as promising red-emitting phosphors under excitation with blue/near UV LEDs.

Among these, in recent years, several red-emitting phosphors doped with Mn^4+^ have been developed. The attention was mainly oriented to fluoride compounds: here, Mn^4+^ shows a narrow emission band near 630 nm, which is about optimal for lighting applications. [4]. On the other hand, some drawbacks (e.g., a need for toxic hydrofluoric acid for their synthesis, poor resistance to temperature and humidity) seriously hamper their development. For this reason, at a recent time, studies have also been addressing other candidate hosts such as oxides and oxyfluorides [2,5].

Among oxides, special attention is paid to aluminates in which Mn^4+^ ions substitute for the Al^3+^ ions in octahedral sites. Mn^4+^ and Al^3+^ have very similar ionic radii but some kind of charge compensation is needed for the stabilization of Mn^4+^ ions in octahedral sites. The properties of Mn^4+^ luminescence in different aluminate hosts are described in several review papers (see, e.g., [2]). In particular, Mn^4+^-doped aluminates whose structures have spinel structured blocks, i.e., the close-packed oxygen layers with Al^3+^ or Al^3+^ and Mg^2+^ in octahedral and tetrahedral sites, were extensively investigated. Such phosphors show luminescence of Mn^4+^ ions within the red spectral range, namely, SrMgAl_10_O_17_ (658 nm) [6], BaMgAl_10_O_17_ (660 nm) [7], Sr_2_MgAl_22_O_36_ (659 nm) [8], Ca_2_Mg_2_Al_28_O_46_ (656 nm) [9], CaMg_2_Al_16_O_27_ (655 nm) [9], Sr_4_Al_14_O_25_ (654 nm) [10], Sr_2_Al_6_O_11_ (652 nm) [11], CaAl_12_O_19_ (658 nm) [12], SrAl_12_O_19_ (658 nm) [13], and LaMgAl_11_O_19_ (663 nm) [14], quite near the edge of eye sensitivity. However, probably the shortest wavelength of Mn^4+^ luminescence in aluminate hosts is observed for the classical ‘spinel’ MgAl_2_O_4_ [15,16,17,18].

Compounds of the spinel group, in particular, the ‘spinel’ itself MgAl_2_O_4_, are well-known matrices for developing phosphors. For instance, the MgAl_2_O_4_ spinel features a cubic structure (*Fd*3*m* space group); in this structure, oxygen anions have a cubic close packing arrangement which creates tetrahedrally and octahedrally coordinated cavities for Mg^2+^ and Al^3+^ cations, respectively [19]. In this structure, Mg^2+^ and Al^3+^ can exchange their positions in the lattice. Therefore, the distribution of cations can be described with the formula (Mg_1-x_Al_x_)[Mg_x_Al_2-x_]O_4_, where *x* is an index expressing the inversion degree. As a result, the spinel crystal structure is disordered, similar to what is observed in solid solutions. Nevertheless, it is generally accepted that this cation inversion allows the fabrication of spinel phosphors where the tetravalent Mn^4+^ ions substitute for Al^3+^ ions. This is obtained by introducing an equivalent concentration of Mg^2+^ ions at the octahedral site. The excess Mg^2+^ ions compensate for the charge unbalance, still maintaining the stoichiometry with respect to oxygen ions in the spinel crystal lattice [15,16].

In our previous studies it was demonstrated that the synthesis parameters strongly affect the luminescence properties of MgAl_2_O_4_ doped with manganese [17,18]. An efficient red-emitting phosphor based on a manganese-doped MgAl_2_O_4_ spinel, with a luminescence peak located at ~651 nm, was prepared by means of a low-temperature annealing phase followed by high-temperature annealing, in an oxidizing atmosphere (air). The low-temperature phase allows for efficient entering of Mn^4+^ in the octahedral sites. The stabilization of Mn^4+^ ions at octahedral sites is obtained, taking advantage of the presence of additional Mg^2+^ ions at octahedral sites, which compensate for the charge unbalance of the lattice structure. Conversely, Mn^2+^ located in the tetrahedral sites produces only pure green (525 nm) emission as observed from a manganese-doped MgAl_2_O_4_ spinel synthesized in a reducing CO atmosphere, even if MnO_2_ is used as a dopant. On the other hand, by using MnO_2_ or Mn_2_O_3_ as dopants, synthesis in neutral argon atmosphere or synthesis without meticulously performed preliminary low-temperature annealing results in the presence of both Mn^2+^ and Mn^4+^ in the spinel phosphor as well as the appearance of the other optical centers showing additional emissions; in particular, some broad-band luminescence located at longer wavelengths in the red region is detected. The nature of this far-red broad-band emission of manganese ions in MgAl_2_O_4_ phosphors remains unclear, thus further investigations are required.

In the present work, the luminescence properties of a series of ceramic spinel MgAl_2_O_4_ phosphors doped with manganese ions and prepared under different synthesis conditions were studied by low-temperature and time-resolved spectroscopy.

## 2. Materials and Methods

The ceramic samples with the spinel lattice structure were synthesized using MgCO_3_ (purity of 99.99%) and Al_2_O_3_ (99.99%) as well as MnO_2_ (99.999%) as a source of manganese ions, by means of a high-temperature solid-state reaction. The starting compounds were weighed to obtain the composition Mg_1.002_Al_1.996_Mn_0.002_O_4_, then mixed in ethanol by using a mortar and a pestle made of agate. The mixture was then dried and uniaxially pressed under about 150 MPa in a stainless-steel die. The resulting pellets were about 2 mm thick and had a diameter of 10 mm. After pressing, the pellets were then placed in a corundum crucible (Thermokeramika, Moscow, Russian Federation). Sample I was subjected to the low-temperature annealing process (consisting of several annealing phases carried out at progressively increasing temperatures, i.e., 500, 600, and 700 °C) and then to the high-temperature annealing process (annealing steps at 1000, 1200, and 1300 °C). Each temperature step lasted for 4 h, in an oxidizing air atmosphere. Conversely, sample II was synthesized without low-temperature annealing. Before each annealing step the tablets were reground and re-pelletized. Moreover, in the last annealing we added to the samples 3 wt% H_3_BO_3_ as flux. One more spinel-structured ceramics doped with manganese ions was prepared by using the mixture of sample II, MgO and MgF_2_ in weighed amounts corresponding to the composition of Mg_1.25_Al_1.75_O_3.75_F_0.25_ (sample III). This mixture was used to obtain pressed pellets which were subjected to annealing at 1200 °C in an argon atmosphere. The sintered pellets of all samples were finally polished for the following characterization. The thermal treatment used for the three types of samples studied in the present work is presented in Table 1.

**Figure 1:**
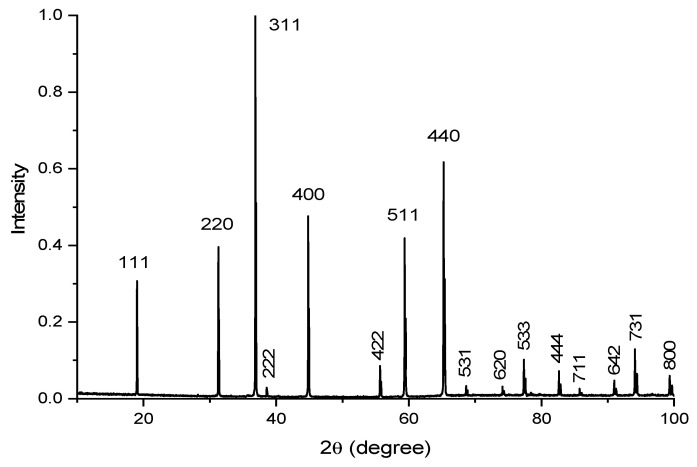
X-ray diffraction pattern of the MgAl_2_O_4_ spinel doped with 0.2 mol% MnO_2_ (sample I). The labels on the peaks are the Miller indices of the corresponding lattice planes.

The structural-phase composition of the obtained ceramic samples was studied using a Bruker D8 Advance X-ray powder diffractometer (Billerica, MA, USA) with monochromatic CuKα radiation, whose voltage and current were set as 40 kV and 40 mA, respectively. XRD data were recorded in the 2θ range from 10° to 100°, with the continuous scan mode at a speed of 0.2 s per step with a step size of 0.02°. Identification of the synthesized compounds was performed with the software package EVA (Bruker) using the ICDD PDF-2 database. The X-ray diffraction analysis confirmed that all the ceramic samples under test had a lattice structure belonging to the cubic system and corresponding to the spinel structure (Figure 1). The unit cubic cell of the various samples had a lattice parameter in the range of 8.07 ÷ 8.09 Å depending on the synthesis conditions.

Time-resolved photoluminescence (PL) spectroscopy was carried out using a JOBIN-YVON SPEX TRIAX 320 spectrometer (Edison, NJ, USA), equipped with a 300 L/mm grating and using an input slit width of 40 μm, resulting in an overall spectral resolution of 0.6 nm. The temperature T of the samples was set in the interval from T~10 K to T~290 K using a CTI-CRYOGENICS optical cryostat cooled (Mansfield, MA, USA) with a closed cycle refrigerator. For the time-resolved studies (luminescence time decay behavior and time-resolved spectra), pulsed laser excitation was used (wavelength 262 nm, pulse duration 10 ns). For this purpose, a frequency quadrupled Nd:YLF laser was used as the excitation source. To reject the ultraviolet radiation scattered by the sample, a long pass filter (made of Schott GG435 glass) was placed in front of the spectrometer entrance slit. The luminescence time decay behaviors were acquired by means of a photomultiplier tube (Thorn EMI 9816QB, Hayes, UK). A digital sampling scope (Tektronix TDS 680B, Heerenveen, The Netherlands) connected to a PC was used to record the decay curves. The spectral acquisition bandwidth was set between 2 and 4 nm by adjusting the spectrometer exit slit width, depending on the signal intensity. The acquisition of the time-gated spectra was carried out using an optical multichannel detector (detector head EG&G 1420, with a controller EG&G OMA2000, Gaithersburg, MA, USA) with image intensification and time-gating capabilities, featuring a spectral resolution (pixel bandwidth) of 0.51 nm. The delay of the time acquisition gate with respect to the laser pulse excitation and the gate width were set by means of using a Stanford DG535 delay generator (Sunnyvale, CA, USA). No correction was applied for the spectral sensitivity of the detector, which decreases very sharply for wavelengths longer than about 730 nm. This feature can remarkably distort the long-wavelength part of the measured spectra. The working spectral span of the multichannel detector EG&G 1420 is ~320 nm. Accordingly, for obtaining PL spectra in the wide spectral range of 350–850 nm, the measurements were performed for two positions of the monochromator corresponding to two values of the central wavelength of the detector spectral span: 650 and 500 nm. Photo-luminescence excitation (PLE) spectra were measured at 300 K using a spectrofluorometer CM2203 (Solar, Minsk, Republic of Belarus).

## 3. Results

The ceramic samples of the manganese ion-doped MgAl_2_O_4_ spinel labeled as sample I were prepared by using MnO_2_ as a dopant and using firstly the annealing in air at low temperatures (steps at 500, 600, 700 °C) and after that the annealing at higher temperatures (steps at 1000, 1200, 1300 °C). The use of this elaborate annealing scheme was motivated by the necessity of obtaining the stabilization of the Mn^4+^ ions at the octahedral site. Indeed, manganese (IV) dioxide begins to decompose at 535 °C to manganese (III) oxide and oxygen [20]. The highest intensity of red emission from Mn^4+^ in MgAl_2_O_4_ was achieved by the final annealing at 1300 °C with the addition of boric acid (H_3_BO_3_) as flux.

**Figure 2:**
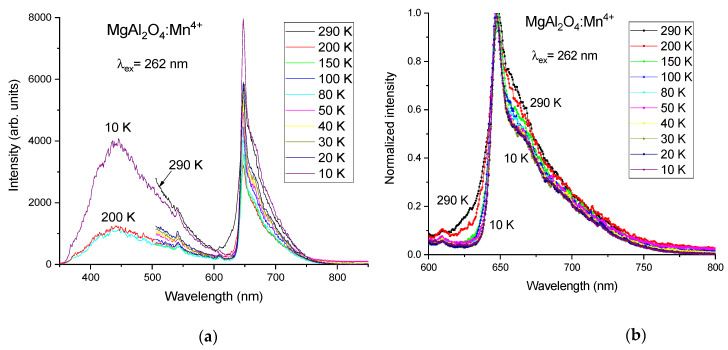
(**a**) PL spectra of MgAl_2_O_4_:Mn^4+^ (sample I) measured at different temperatures: 290, 200, 150, 100, 80, 50, 40, 30, 20, and 10 K; (**b**) PL spectra of MgAl_2_O_4_:Mn^4+^ (sample I), normalized at their peak value, for the red spectral region measured at different temperatures.

When excited at 262 nm wavelength, the PL spectrum of sample I, see Figure 2a, is dominated by a relatively broad emission in the red, with a narrow peak at 651 nm. The full-width at half maximum (FWHM) of this peak is ~40 nm at 290 K. According to many previous publications, the observed red emission corresponds well with the luminescence of Mn^4+^ [15,16,17,18], although a broad-band emission in the blue-green peaking at 430 nm is also observed under 262 nm excitation wavelength. The measurements in the short-wavelength region were performed only for three selected temperatures: 200, 80, and 10 K. The 430 nm band is not detected under Mn^4+^ intracenter excitation in the near UV/blue spectral region as was shown in previous publications [17,18]. Since this 430 nm emission is observed from all the synthesized samples of MgAl_2_O_4_, doped with manganese ions, under 262 nm excitation wavelength, it can tentatively be attributed to some kind of spinel defect-related emission. Accordingly, it is not relevant for practical applications in LEDs, where the excitation occurs in the blue or near UV spectral range.

Under cooling, the PL spectrum of sample I becomes narrower and the short-wavelength wing of the spectrum practically disappears at 10 K, see Figure 2b. Due to the small relative movements of the sample with respect to the laser beam and collection optics during the cooling phase, affecting the signal collection efficiency, it was not possible to compare the absolute luminescence intensity at different temperatures in Figure 2a. For this reason, in Figure 2b, the spectra normalized to the maximum intensity of the peak at 651 nm are presented for the most important red spectral region.

**Figure 3:**
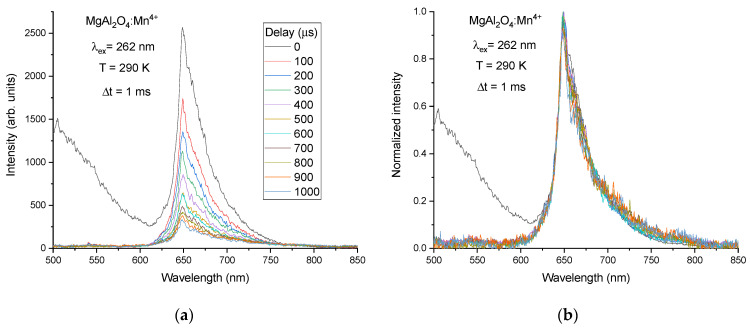
(**a**) Time-resolved PL spectra of the spinel MgAl_2_O_4_ ceramics doped with 0.2 mol% MnO_2_ (sample I) measured at 290 K with different delays (0–1 ms) between the laser pulse and the time gate (Δt = 1 ms); (**b**) the same spectra normalized to the peak intensity.

The time-resolved PL spectra from sample I have been measured at a room temperature with time delays between 0 and 1 ms and with a time gate width of 1 ms, see Figure 3. The spinel defect-related broad-band emission disappears already at the shortest delay value 0.1 ms, i.e., this emission has a rather fast decay and does not influence the PL spectrum at longer delays. The decay time of this defect-related emission is estimated to be ~5 µs. Due to this reason, the time-resolved PL spectra were not studied in the spectral range shorter than 500 nm with the position of central wavelength of the detector at 500 nm. The normalized time-resolved PL spectra show that the shape of PL spectrum does not change with the time delay, i.e., luminescence within the whole PL spectrum decays with the same decay law and accordingly corresponds to the decay of the same emitting state.

**Figure 4:**
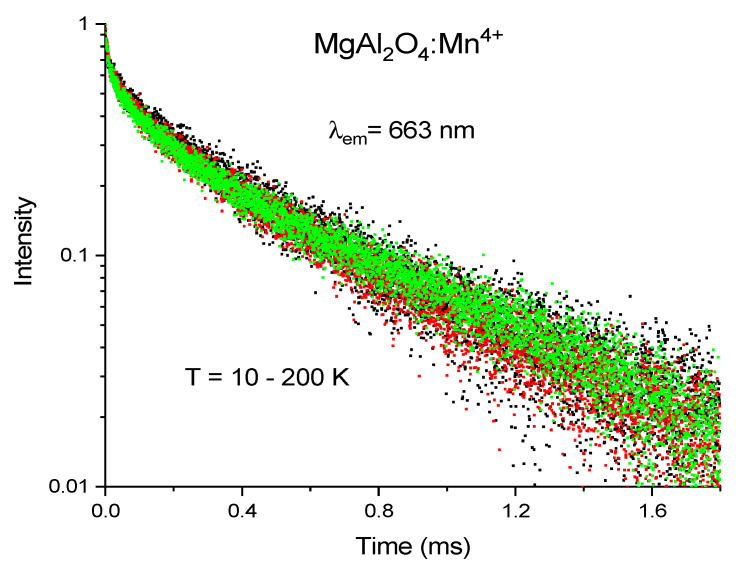
Normalized decay curves of red (λ_em_ = 663 nm) luminescence recorded from sample I, MgAl_2_O_4_ doped with 0.2 mol% MnO_2_, at T from 10 K to 200 K.

Decay curves of the red luminescence acquired at different values of temperature after pulsed excitation at 262 nm show non-exponential behavior, see Figure 4, which indicates the presence of some kind of extrinsic quenching of the luminescence, probably due to the energy transfer from Mn^4+^ ions to some quenching centers originated by defects. The obtained decay curves were fitted by a double exponential decay function. The value of the longer decay component, which can be considered an estimation of intrinsic radiative decay time, is ~0.4 ms at 290 K and practically it does not change with temperature.

**Figure 5:**
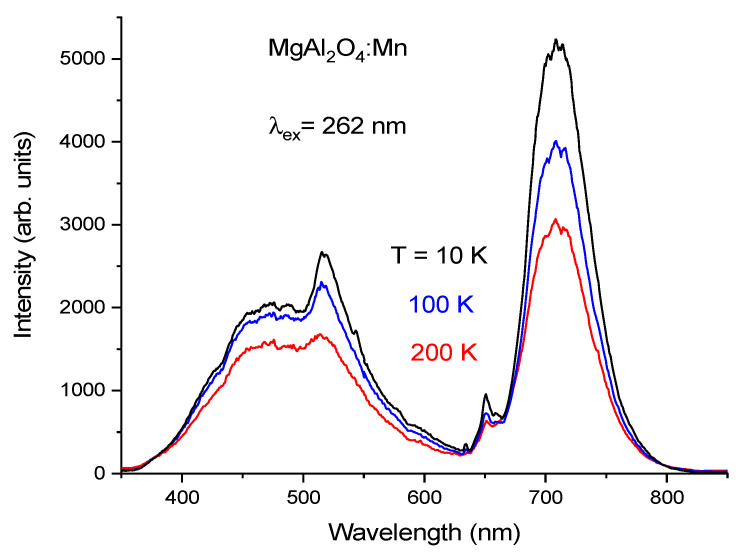
PL spectra of sample II, i.e., MgAl_2_O_4_:Mn, measured at different temperatures: 200 K, 100 K, and 10 K.

**Figure 6:**
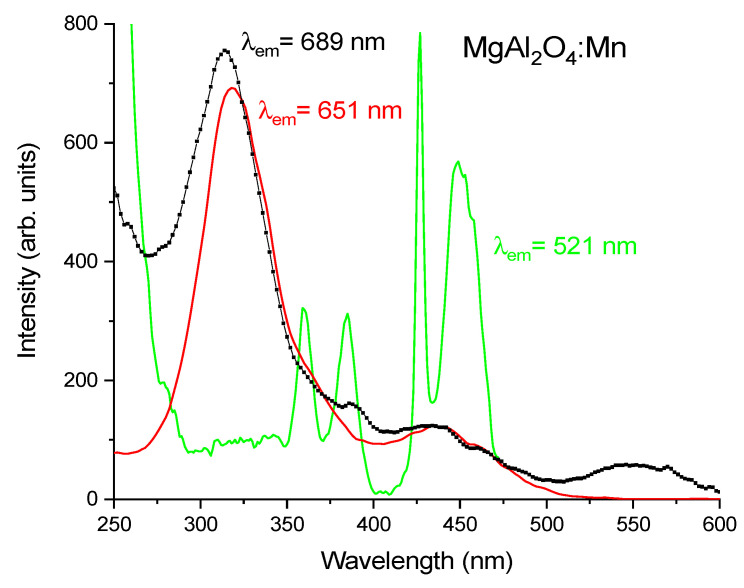
PLE spectra of sample II, i.e., MgAl_2_O_4_:Mn, measured at 290 K by monitoring different emission wavelengths.

On the other hand, in MnO_2_ doped MgAl_2_O_4_ samples subjected to the high temperature annealing only (temperature steps at 1000, 1200, and 1300 °C) but without the low temperature preliminary annealing phase (sample II), the luminescence of Mn^4+^ ions in the red at 651 nm practically disappears: in the red region the PL spectrum is mainly constituted by the broad-band far-red luminescence band having a maximum between 690 ÷ 710 nm; moreover, an additional green luminescence peak appears at 520 nm, see Figure 5. As it is well-known, the luminescence of manganese ions doped MgAl_2_O_4_ in the green is due to the ^4^T_1_ → ^6^A_1_ transition of Mn^2+^ ions occupying the tetrahedral sites by substituting for Mg^2+^ ions in the spinel [15,21]. The PLE spectrum of Mn^2+^, reported in Figure 6, has a characteristic shape featuring four clearly distinguishable bands in the range 350 ÷ 470 nm. One of these bands, typically located near 400 nm (in our case at 430 nm) is rather narrow. The increasing intensity of the green emission at wavelengths shorter than 290 nm is not due to an increase of the Mn^2+^ luminescence intensity but is caused by the appearance of the broad-band defect-related emission, whose PL spectrum overlaps with that of Mn^2+^ luminescence. The latter circumstance does not allow studying the spectral properties of Mn^2+^ luminescence under 262 nm laser excitation separately from those of this defect-related emission.

We note that the PLE spectrum of the emission band in the red with maximum at 651 nm (see Figure 6, red line) has two excitation bands in the blue (peaking at 446 nm) and near UV (peaking at 364 nm). Referring to the scheme of the energy levels of Mn^4+^ provided by the Tanabe-Sugano (TS) diagram for the d^3^ electron configuration under an octahedral crystal field (CF) [22], these bands can be attributed to the spin-allowed transitions of Mn^4+^, namely, ^4^A_2_ → ^4^T_2_ and ^4^A_2_ → ^4^T_1_, respectively. Additionally, the observed excitation band with a peak near 325 nm is due to the O^2-^—Mn^4+^ charge-transfer transition. On the other hand, according to Figure 6, the PLE spectrum of broad-band far-red emission coincides with that of Mn^4+^ luminescence in the range of 300 ÷ 500 nm, i.e., in the region of Mn^4+^ strong absorption on spin-allowed d-d and fully allowed charge-transfer transitions. Some difference in PLE spectra is observed at wavelengths shorter than 300 nm, in particular, around the excitation laser wavelength and in the range of λ > 500 nm.

**Figure 7:**
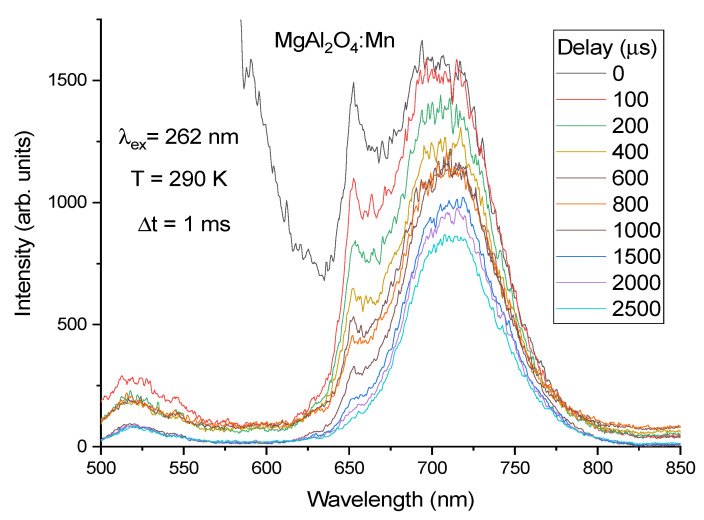
Time-resolved PL spectra of MgAl_2_O_4_:Mn (sample II) measured at 290 K at different delays (0 ÷ 2.5 ms) of the time gate (Δt = 1 ms) with respect to the laser pulse.

When the temperature increases from 10 K to 200 K, the intensities of both the broad-band far-red luminescence as well as Mn^2+^ green emission decrease but not so strongly as that of Mn^4+^ luminescence, traces of which can also be seen near 650 nm in the PL spectrum of sample II (Figure 5). The decay time of the broad-band far-red luminescence at 290 K has been estimated to be τ~4.0 ms. Time-resolved PL spectra measured for sample II in the time delay range of 0 ÷ 2.5 ms confirm that the broad-band far-red luminescence has a longer decay time than Mn^4+^ luminescence and that the latter decays completely at longer time delays (see Figure 7). As in the case of sample I, the spinel defect-related emission disappears from the spectrum of sample II at the shortest time delay of 0.1 ms, and the Mn^2+^ luminescence band at 520 nm can be better recognized in the time-resolved PL spectra where this luminescence is still observable at long time delays (decay time is in the order of several ms). Again, as in the case of sample I, the time-resolved PL spectra were not studied in the spectral range shorter than 500 nm.

**Figure 8:**
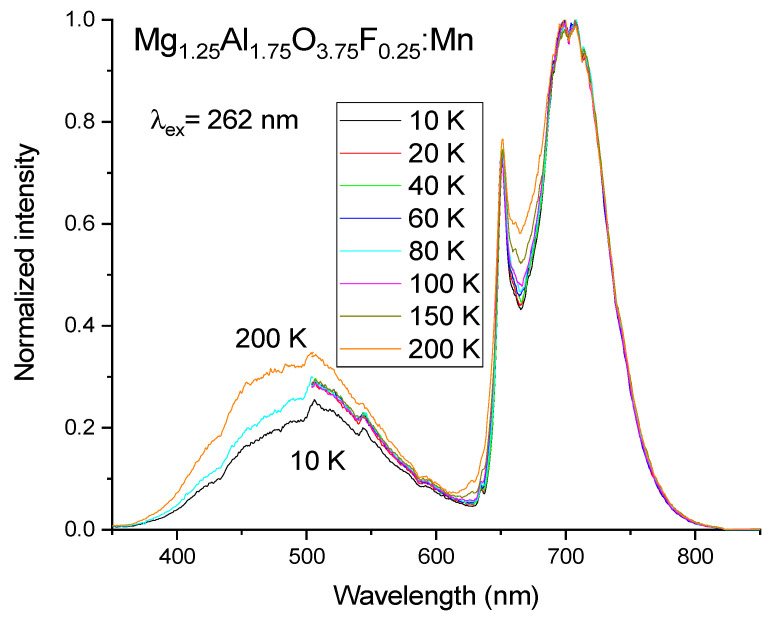
Normalized PL spectra of manganese ions doped Mg_1.25_Al_1.75_O_3.75_F_0.25_ (sample III), measured at different temperatures.

One more peculiarity of magnesium aluminate spinels doped with manganese ions is that the increasing ratio of Mg^2+^ to Al^3+^ results in the magnification of the Mn^4+^ luminescence intensity even if the synthesis of such spinels is carried out in a neutral argon atmosphere. Figure 8 reports the luminescence spectra of manganese ions doped Mg_1.25_Al_1.75_O_3.75_F_0.25_ spinel (sample III) synthesized in argon by using MgAl_2_O_4_:Mn (sample II) as a starting material. The measurements in the short-wavelength region were performed only for three selected temperatures: 200, 80, and 10 K. One can see that in sample III, the Mn^4+^ luminescence intensity relative to the intensity of the broad-band far-red luminescence is more intense than in sample II, taking into account that the intensities of the broad-band far-red luminescence in these samples have practically the same values. It should also be noted that the ratio of the intensities for the luminescence bands at 651 and 700 nm for sample III is almost constant in the range of temperature from 10 K to 200 K.

**Figure 9:**
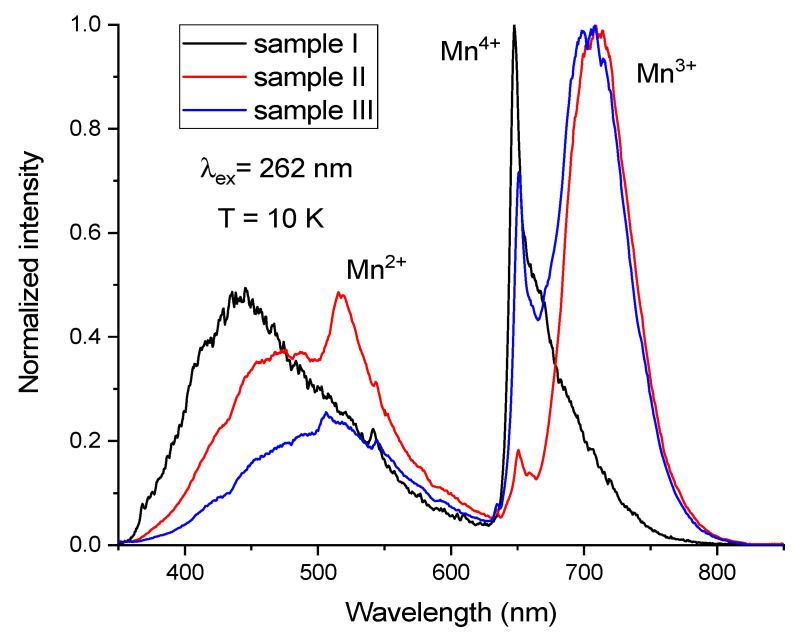
Normalized PL spectra of samples I, II, and III measured at 10 K.

In order to demonstrate the difference between the position and relative intensities of the different bands, the PL spectra of all three samples measured at 10 K are presented in one graph in Figure 9.

## 4. Discussion

The observed low-temperature and time-resolved properties of the red emission band in the PL spectrum of sample I correspond well to those of Mn^4+^ luminescence from the MgAl_2_O_4_ spinel studied earlier at room and higher temperatures [15,16,17,18]. In particular, the narrow feature of the band at 651 nm is the zero-phonon line (ZPL) of the Mn^4+ 2^E → ^4^A_2_ transition, while the bands of the PL spectrum at longer and shorter wavelengths correspond to Stokes and anti-Stokes vibronic sub-bands, respectively. All these PL spectrum features show inhomogeneous broadening due to the cation disorder caused by inversion in the spinel MgAl_2_O_4_ structure. In particular, the disorder leads to spectral smearing of vibronic lines. This interpretation is confirmed by the results of the low-temperature measurements which have shown that the main peak at 651 nm considerably narrows with decreasing temperature and the short-wavelength wing of the PL spectrum, i.e., the anti-Stokes vibronic side-band disappears at low temperatures. The time-resolved PL spectra of sample I have shown that all the components of the spectrum are due to a decay from the same initial emitting state, i.e., to the ^2^E → ^4^A_2_ transition in Mn^4+^ including the vibronic transitions. Thus, it can be concluded that the synthesis procedure applied to sample I provides stabilization of practically all manganese ions in the tetravalent oxidation state.

The obtained properties of Mn^2+^ green luminescence under 262 nm laser excitation also correspond well to those of Mn^2+^ in different spinel structures studied earlier although overlapping of the Mn^2+^ emission spectrum with the spectrum of some spinel defect-related emission does not allow studying these properties in detail. Besides that, the efficiency of Mn^2+^ luminescence excitation is lower when these samples are excited at 262 nm than in the case of lower-energy excitation.

Some hypotheses can be proposed for the explanation of the nature of the broad-band emission at 675 ÷ 720 nm which is observed in MgAl_2_O_4_:Mn phosphor samples. In particular, broad-band luminescence of Mn^4+^ ions can be observed instead of the narrow-band emission in the case of weak CF strength. The luminescence properties of d^3^ ions (Mn^4+^) in octahedral coordination are determined by transitions from the states ^2^E and ^4^T_2_ (i.e., the two lowest-energy excited states); moreover, the energy of the ^4^T_2_ level is strongly affected by the CF strength. Indeed, the CF strength determines the position of the ^4^T_2_ level with respect to the ^2^E state as well as the crossover point for these levels in the d^3^ TS diagram. For this reason, in materials where Mn^4+^ ions are affected by a strong CF, the narrow-band Mn^4+^ luminescence associated with the transition ^2^E → ^4^A_2_ is observed, but in materials with crystallographic sites inducing weak CF, the broadband ^4^T_2_ → ^4^A_2_ emission can be observed from Mn^4+^. However, the latter transition is spin-allowed, i.e., such luminescence should show a relatively fast decay, at least a faster decay than that obtained in our studies for this band (τ ~ 4.0 ms). It could be suggested that the value of the CF strength is near the crossover point for ^2^E and ^4^T_2_ levels, i.e., these levels are thermally coupled, but in this case, the ratio of intensities of the 651 nm peak and this broad band should strongly change with temperature, which is not observed. Besides that, it is unreasonable to expect that in phosphors based on the same host but obtained under different synthesis conditions Mn^4+^ ions can occupy octahedral sites inducing such different crystal fields: either strong CF (sample I) or weak CF (sample II).

Another hypothesis is that this broad-band emission in the far-red originates from the luminescence of Mn^2+^ ions located in octahedral sites. This explanation is proposed, for instance in [23] where the broad-band emission in the far red from the defect-rich MgAl_2_O_4_ spinel doped with Mn ions is attributed to luminescence of Mn^2+^ centers in the octahedral sites. If Mn^2+^ substitutes for Al^3+^ in the octahedral sites the charge compensation can be reached through inversion without introducing other ions. According to the TS energy-level diagram for d^5^ electron configuration, luminescence of Mn^2+^ ions can be observed in significantly different spectral ranges depending on the strength of CF affecting these ions. In particular, luminescence in the green spectral range is expected for Mn^2+^ ions entering the tetrahedral sites whereas red and even NIR luminescence can be observed from Mn^2+^ occupying octahedral sites. It should be noted here that in the case of the d^5^ electron configuration, the TS diagrams for octahedral and tetrahedral coordination are identical by taking into account that the CF strength is stronger for octahedral sites than for tetrahedral ones.

As all the Mn^2+^ absorption transitions are spin-forbidden; the corresponding bands in the PLE spectrum of Mn^2+^ can be concealed by the wider and more intense bands caused by spin-allowed transitions within Mn^4+^. In this case the PLE spectra of such possible far-red Mn^2+^ luminescence and the red Mn^4+^ luminescence in the blue and near UV regions can coincide if one considers that the far-red luminescence from Mn^2+^ is excited by the energy transfer from Mn^4+^ ions to Mn^2+^ ions. This similarity of PLE spectra is indeed observed in the experiment for the broad-band far-red luminescence and the red Mn^4+^ luminescence. On the other hand, the PLE spectrum of 520 nm emission corresponding to the luminescence of Mn^2+^ entered into the spinel tetrahedral sites is well reproduced with all its specific features, see Figure 6. Therefore, it is unclear why the respective features are not seen in the PLE spectrum of the observed far-red luminescence if this far-red luminescence originates from the Mn^2+^ ions entered into octahedral sites. Besides that, one could hardly suggest that Mn^2+^ ions having an ionic radius of 0.83 Å in octahedral coordination can substitute for Al^3+^ ions having an ionic radius of 0.535 Å in the same one [24], i.e., the ionic radii of these octahedrally coordinated ions differ by more than 50%, and according to standard crystallographic rules, such substitution is highly unlikely by taking into account that the ratio of Mg^2+^ and Mn^2+^ ionic radii in tetrahedral coordination is 0.57/0.66 Å. In other words, it is most likely that in the spinel MgAl_2_O_4_ structure, the Mn^2+^ ions occupy the tetrahedral sites but not the octahedral ones.

Generally speaking, it can be assumed that in compounds doped with Mn ions, the octahedral sites contain both Al^3+^ as well as Mn^3+^ ions, for which charge compensation is not required. Information on the luminescence behavior of Mn^3+^ is very limited [25,26]. In general, it is expected that in the majority of the hosts, the Mn^3+^ luminescence is quenched due to the large Jahn-Teller splitting of Mn^3+^ energy levels. Considering the TS diagram for d^4^ ions, the broad-band luminescence of Mn^3+^ entering into the octahedral site can be due to either the spin-allowed ^5^T_2_ → ^5^E transition which has typically rather short decay time (of the order of tens μs [26]) or the slower spin-forbidden ^1^T_2_ → ^5^E transition, depending on the CF strength. The Mn^3+^ broad-band absorption is due to the strong spin-allowed ^5^E → ^5^T_2_ transition, typically located at 500 ÷ 550 nm, i.e., just in the spectral range where some additional band in the PLE spectrum of emission at 689 nm is observed for sample II, see Figure 6. In the spectral range 325 ÷ 500 nm, absorption by Mn^4+^ ions dominates and the shape of the PLE spectrum of the broad-band far-red luminescence almost coincides with that of Mn^4+^ luminescence monitored at 651 nm, which can be simply explained by the overlapping of emission bands of these two kinds of luminescence centers but can be partly due to the presence of the energy transfer from Mn^4+^ to Mn^3+^. However, at wavelengths shorter than ~325 nm, the broad-band far-red luminescence is excited more efficiently, which can be due to some kind of O 2p-Mn 3d charge-transfer transition. The decay time of this broad-band far-red luminescence is rather long (i.e., τ ~ 4.0 ms) and so this luminescence can be ascribed to the Mn^3+^ spin-forbidden ^1^T_2_ → ^5^E transition. In the presence of charge compensation by extra amounts of Mg^2+^ ions, the efficient conversion of Mn^3+^ to Mn^4+^ takes place, resulting in the appearance of Mn^4+^ luminescence in sample III and in the complete disappearance of this broad-band far-red luminescence in sample I synthesized under oxidation conditions.

## 5. Conclusions

The obtained low-temperature and time-resolved features of the red emission band characterizing the PL spectrum of the Mn^4+^-doped MgAl_2_O_4_ sample confirmed the generally accepted model of this luminescence as caused by the Mn^4+ 2^E → ^4^A_2_ transitions, including the ZPL located at 651 nm and the Stokes and anti-Stokes vibronic side-bands, which are broadened by the cation disorder caused by inversion in the spinel crystal structure. In this work it has been shown that the special multi-step annealing procedure applied for the solid-state synthesis of a MgAl_2_O_4_:Mn^4+^ phosphor provides the stabilization of practically all manganese ions introduced into the phosphor in the tetravalent state. Furthermore, red-emitting MgAl_2_O_4_:Mn^4+^ phosphor synthesized by this method demonstrates good color characteristics (CIE 1931 color coordinates are x = 0.72; y = 0.28 [18]); it can be therefore considered as promising red phosphor for the realization of warm pc-WLEDs. The broad-band far-red luminescence observed from MgAl_2_O_4_ phosphors containing manganese ions non-stabilized in the tetravalent state has been attributed to spin-forbidden ^1^T_2_ → ^5^E transitions of Mn^3+^ ions substituting for Al^3+^ ions in the octahedral sites in the spinel structure. Such MgAl_2_O_4_:Mn^3+^ phosphors can be used for different lighting applications, among them those related to the agricultural lighting [27].

## Figures and Tables

**Table 1:** Experimental conditions applied for the synthesis of ceramic spinels containing manganese ions.

Sample Label	Host	Atmosphere	Annealing Temperature (°C)
Sample I	MgAl_2_O_4_	air	500, 600, 700 and 1000, 1200, 1300
Sample II	MgAl_2_O_4_	air	1000, 1200, 1300
Sample III	Mg_1.25_Al_1.75_O_3.75_F_0.25_	argon	1200

## Data Availability

Data is contained within the article. Further numerical data (e.g., spectra shown in Figures) are available on request from the corresponding Author.
